# Climate mitigation potential of natural climate solutions and clean energy on The Nature Conservancy properties in California, USA

**DOI:** 10.1371/journal.pone.0311195

**Published:** 2024-10-21

**Authors:** Kristen N. Wilson, Daniel W. Salzer, Michelle C. Passero

**Affiliations:** 1 The Nature Conservancy, San Francisco, California, United States of America; 2 The Nature Conservancy, Portland, Oregon, United States of America; University of Ferrara, ITALY

## Abstract

Natural climate solutions (NCS) and transitioning to clean energy can reduce greenhouse gases and contribute to mitigating climate change. Private landowners with large holdings, such as conservation organizations like The Nature Conservancy, have set ambitious goals to reduce net emissions and increase sequestration on their lands by implementing NCS. We assessed the potential carbon dioxide-equivalent (CO2e) reduction from feasible NCS, specifically implementing new restoration and agricultural management activities, and transitions to clean energy on The Nature Conservancy, California chapter’s fee-owned and conservation easement properties. We compared the total CO2e reduction from potential new NCS activities to the impact from ongoing NCS activities, the chapter’s 2030 goal, and the state’s reduction goal for natural and working lands to understand how the organization can contribute to climate mitigation. We found that implementing NCS on 37 fee-owned properties (63,175 MTCO2e year –1) and clean energy on 10 fee-owned properties (488 MTCO2e year –1) combined would not reach the chapter’s 2030 goal (72,000 MTCO_2_e year –1), and there can be tradeoffs between maximizing CO2e reduction and protecting conservation values. However, ongoing changes to forest management on a single conservation easement property, where another non-profit harvests timber and sells carbon credits, currently contributes 147,749 MTCO2e year –1, more than two times the 2030 goal and representing 7.4% of the state’s annual goal. Our results suggest that The Nature Conservancy, California chapter would need to implement NCS on some of the conservation easements or consider future land protection deals with carbon rich ecosystems or high impact NCS to reach their CO2e reduction goal.

## Introduction

Implementing natural climate solutions (NCS) and transitioning to clean energy are essential to reduce greenhouse gas emissions to, and increase removal from, the atmosphere. Combined, these actions can help to mitigate the climate crisis. NCS are land management activities that increase carbon sequestration and storage or avoid greenhouse gas emissions by protecting, restoring, or improving land management [1]. NCS include protecting existing ecosystems from conversion, restoring ecosystems, and improving agricultural or forest management. While NCS show great promise at global [2], national [1], and state-level scales [3–5], they have not been widely implemented to date. Most NCS projects have involved tree planting and reforestation, primarily in places where mitigation funds exist to pay for activities or where action is taken by private sector corporations [6].

Opportunities for NCS projects on large, contiguous swaths of public lands may be relatively easy to envision, if not to enact. On private lands, however, coordinated implementation of NCS, as with any cohesive land management, may be hampered by small and fragmented parcels and the presence of many landowners with various needs and interests. To make progress towards NCS implementation, The Nature Conservancy, California chapter which is the largest private conservation land manager in the state including 72,439 ha held in fee as the landowner and 140,831 ha in conservation easements as of 2020, set a goal that year to reduce net emissions and increase sequestration in the state using NCS by 720,000 metric tons of carbon dioxide-equivalents (MTCO_2_e) by 2030 [Dick Cameron, personal communication].

The basis for the 2030 goal was that ten percent of lands owned in fee by The Nature Conservancy in California (7,244 ha) would have NCS implemented, specifically restoration and changes to agricultural activities, with an assumed 10 MTCO2e ha^-1^ year ^-1^ sequestered for an annual rate of 72,000 MTCO_2_e year ^-1^ over a decade. The Nature Conservancy, California chapter set this goal based on fee-owned lands because it is much easier to implement NCS and clean energy as a landowner compared to implementing these activities on a conservation easement. A conservation easement is a voluntary, legal agreement between a landowner and an organization, typically a land trust or government agency, to protect conservation values by limiting the landowner’s rights to develop or use their property. The landowner, and any future landowners, can use the property within the limits of the easement terms. While conservation easement can be a helpful legal instrument to implement and create incentives for NCS, the easement terms were already executed at a time when the climate benefits of NCS were not contemplated. Any future implementation of NCS with a conservation easement or on a property with an existing conservation easement would require the landowner’s consent.

Implementation of NCS on 10% of The Nature Conservancy’s fee-owned lands seemed like a reasonable target to aim for over a decade, and the sequestration rate was based on science at the time showing ≥10 MTCO_2_e year –1 for four out of five NCS restoration activities [3]. The goal did not include two NCS activities, changes to forest management or avoided conversion (i.e. land protection), because these activities apply to new property deals, not existing properties. Changes to forest management, specifically shifting from even aged to selective harvest, are made by default when the chapter acquires industrial timberland property or sets easement terms to conserve biodiversity alongside timber harvest.

To date, The Nature Conservancy, California chapter has implemented or been associated with three NCS projects. The first offset project was the Garcia River Forest property, where changes to redwood timber harvest commenced in 2004. The Conservation Fund, another environmental non-profit, owns and manages the 9,623-ha timberland. The Nature Conservancy helped acquire the property and holds a conservation easement which specified that The Conservation Fund had to gain certifications from the Forest Stewardship Council and Sustainable Forest Initiative Standard and establish a timber Management Plan. The Conservation Fund sells carbon credits in the regulatory marked for forest harvest practices that increase carbon storage, and the recent 2020–2022 reduction rate was on average 147,749 MTCO2e year^-1^ [7]. The second project is Childs Meadow, a wetland restoration project funded by Cap-and-Trade Greenhouse Gas Reduction Funds implemented in 2016 that sequesters 38 MTCO2e year^-1^ across 7.5 hectares [8]. The third project at Staten Island in the California Delta will be registered under the American Carbon Registry (ACR581) in 2025 for crop conversion from corn to rice (1,671 ha) and wetland restoration (176 ha) which are being implemented from 2019–2025 with an average reduction of 11,180 MTCO2e year^-1^ once fully implemented [9].

In this study, we assessed the CO2e reduction potential from realistic ecosystem and infrastructure-based consideration of feasible NCS activities and transitions to clean energy on The Nature Conservancy fee and easement lands in California. We included transitions to clean energy on these lands plus offices because phasing out the use of fossil fuels is a primary climate mitigation strategy, and like NCS can be implemented by a landowner. We compared the total CO2e reduction from the potential NCS and clean energy on fee lands where TNC is the landowner to the three ongoing NCS projects, the chapter’s 2030 reduction goal, and the state’s ambitious reduction goal of 2 million MTCO2e year^-1^ from 2020–2030 for natural and working lands [10]. Based on fee land ownership only, The Nature Conservancy is the fourth largest private landowner in the state, behind two industrial timber companies and The Wildland Conservancy, another non-profit land conservation organization. By downscaling the potential climate mitigation to a single, large landowner, the contribution that the chapter can make to their 2030 goal, and tradeoffs that can limit the carbon mitigation potential become clear. This approach could be duplicated by other landowners to determine their individual climate mitigation potential based on NCS or clean energy transitions.

## Methods

We surveyed fifteen stewardship and science staff from the chapter to determine the feasibility of eight NCS activities in California. We presented a photo of each NCS activity along with a brief description of the activity and asked if that staff thought it made sense to implement this activity on some of The Nature Conservancy’s fee and conservation easement lands (S1 Fig). Participants scored their response from 1–5; (1) strongly disagree, (2) disagree, (3) neither agree nor disagree, (4) agree, and (5) strongly agree. We calculated the mean score and response rate for each NCS activity (S1 Table). Based on the feasibility survey and follow-up conversations with staff, we eliminated *compost application* which was deemed to be unsuitable because it could negatively impact biodiversity by introducing non-native seeds.

Past studies are mixed on the impact of compost application on biodiversity finding it can promote invasive species [11], not affect plant diversity [12], or have been inconclusive on impacts to attributes of plant diversity important for biodiversity conservation [13]. Given the uncertainty and what one survey respondent said was a “very controversial practice” there is a need for additional study on the impact of this NCS on biodiversity. Another respondent noted there is almost always plastic trash in compost that could have a negative impact on sensitive species. The Nature Conservancy, California chapter’s position has been that compost could be appropriate for highly disturbed lands such as agricultural fields, but more research is needed for application in other areas.

We identified properties where the feasible NCS activities could be applied using multiple sources. We relied on past studies [5, 14] to determine where *agroforestry*, *cover cropping*, and *riparian restoration* applied (Table 1), and then used a vegetation dataset [15] to calculate the total possible area for each activity on each property (Table 2). Marvin et al. [5, 14] modeled NCS under different climate change projections and implementation rates: agroforestry was implemented on ~3,198 ha year^-1^ (7% of all agricultural land annually), cover cropping on ~69,000 ha year^-1^ (three times the recent 2001–2014 annual rate of cover cropping), and riparian restoration on ~2,000 ha year^-1^ (25% greater than the annual rate of total potential riparian restoration in the Central Valley). We used data from these two studies for the 2020–2050 period, to not limit the area available for NCS activities, under an RCP 8.5 emissions trajectory and the CanESM2 ‘average’ climate model. We further refined the area of *riparian restoration* on the fourteen properties identified by using aerial imagery to delineate riparian buffers based on existing riparian corridors that were visible upstream or downstream. We did this to avoid limiting the treatment areas based on a set 30 m riparian buffer width being applied to streams of different sizes. *Agroforestry* covered 148 ha, *cover cropping* 4,292 ha, and *riparian restoration* 723 ha on The Nature Conservancy, California chapter lands.

**Table 1:** Natural climate solutions included in the feasibility survey. Descriptions of the activities are the same as presented in the survey. Compost application was deemed not feasible because of possible conflicts with biodiversity conservation and woodland restoration is not included as we did not find any studies on the sequestration rate for oak restoration.

Activity		Activity Description	Spatial Data Source	Spatial Data Description
Agroforestry		Plant trees along field boundaries.	[5, 14]	Margins of agricultural land, Central Valley only.
Cover cropping		Plow under cover crop prior to cash crop planting.	[5, 14]	Agricultural land.
Rice BMPs		Farming practices to reduce emissions.	[15]	Rice fields.
Riparian restoration		Establishing forest cover along streams.	[5, 14]	Agricultural land and grasslands adjacent to major waterways. Existing forest cover masked out within stream buffer.
Urban tree reforestation		Planting trees.	[17]	Urban canopy cover in areas >90 m^2^, excluding impervious surfaces.
Wetland restoration	Cultivated to freshwater wetland	Restoration of lands that were formerly peatland back to wetlands.	[17–21]	Hydric soils, herbaceous vegetation and nonperennial croplands, not including rice fields or existing wetlands
	Pasture to freshwater wetland			
	Cultivated to tidal wetland	Restoration of tidal wetlands.	[22]	Undeveloped upland wetland habitat projected to be inundated by sea level rise or adjacent o vulnerable coastal habitat.
	Pasture to tidal wetland			

**Table 2:** Feasible natural climate solutions applied to specific vegetation types.

Vegetation Type	Agroforestry	Cover cropping	Riparian restoration
Annual Grassland			X
Barren			X
Blue Oak Woodland			X
Cropland	X	X	X
Deciduous Orchard	X		X
Dryland Grain Crops	X	X	X
Irrigated Grain Crops	X	X	X
Irrigated Hayfield	X	X	X
Irrigated Row and Field Crops	X	X	X
Pasture			X
Perennial Grassland			X
Rice			X
Vineyard	X	X	

*Urban tree restoration* was possible in developed areas and developed open space [16], not including impervious areas, in places where the area for planting was >90 m^2^ [17]. This NCS activity covered 97 ha. *Freshwater wetland restoration* areas consisted of herbaceous vegetation [18] and nonperennial croplands not including rice fields [19] with historic hydric soils indicative of wetlands [20] and excluding existing wetlands [21]. *Tidal wetland restoration* areas were undeveloped uplands and levees in existing wetlands that were either projected to be inundated from sea level rise or were adjacent to vulnerable habitats [22]. For freshwater and tidal wetland restoration, we determined whether the property was cultivated or pasture using the same vegetation dataset used for *agroforestry*, *cover cropping*, and *riparian restoration* [15]. *Freshwater wetland restoration* covered 4,785 ha and *tidal wetland restoration* covered 594 ha, both types of restoration applied primarily to cultivated agricultural fields and secondarily to pastures. *Rice best management practices* was identified in rice fields and covered 351 ha.

We assumed that the NCS activities were not mutually exclusive, meaning they could co-occur at the same location. This assumption does not hold for seven properties where wetland restoration overlapped spatially with agricultural management activities: *agroforestry*, *compost application*, *cover cropping*, and *rice best management practices*. To understand the impact of the overestimate, we calculated what the total CO2e reduction would be for the seven properties if the greater reduction activity, wetland restoration or agricultural NCS only (combined total of all agricultural activities) was implemented and compared this to the total reduction with the overlapping wetland and agricultural activities.

We compiled information on CO2e sequestration rates for all feasible NCS activities using a systematic literature review following the PSALSA approach [23]. We searched Google Scholar from 2015, when the state implemented a Greenhouse Gas Reduction Fund to implement and study NCS, to 2023 using specific search strings (S3 Table). To refine our search terms, we first did a pilot search. Articles that were in peer-reviewed journals, in English, accessible, specific to California, similar to the NCS activity descriptions, and primary research were included in the screening process. The initial studies found were reduced based on the screening criteria and we evaluated the first fifty results as the studies beyond that number did not match any of the inclusion criteria. Two activities, *agroforestry* and *rice best management practices*, had no California specific studies so we relied on meta-analysis from other states. We removed *woodland restoration* as we did not find any studies on the change in carbon sequestration or emissions with oak restoration in the literature, even outside of California.

We recorded the sequestration rates from the studies, calculated the mean value, and used the range of mean values from individual studies to represent the uncertainty. For the activities with only one study, we used the standard error or 95% confidence interval to represent the uncertainty. We converted Mg C to CO2e using a conversion factor of 3.67, the molecular ratio of carbon dioxide to carbon (44 g mole^-1^CO_2_) / (12 g mole^-1^CO). We combined carbon sequestration in biomass and soils when they were reported separately, and only included empirical values [24]. For studies that reported total carbon sequestration over a multi-year period we calculated the mean annual rate assuming a linear increase. While carbon accumulation shows a sigmoidal growth over time for riparian restoration [25], the increase over the first fifty years for ‘riparian woodlands,’ the type of riparian vegetation in this study, was linear.

We calculated the CO2e reduction (MTCO2e year –1) rate by multiplying the sequestration rate (MTCO2e ha^–1^ year –1) by the area of each NCS activity (ha) on individual properties. For ongoing NCS activities at Staten Island we calculated CO2e reduction using the mean sequestration rate for *cover cropping* and *cultivated to managed wetland restoration* from the literature review. We assumed the mean sequestration rate annually did not decrease or saturate from 2020–2030. *Changes to forest management* does begin to result in saturation of mitigation at 25 years [1] and would apply to the Garcia River Forest property after 2032. The other activities all reach saturation after more than forty years, beyond the 2030 goal period [1].

Clean energy solutions to reduce emissions was based on data from the consumption of electricity and propane to power buildings and water pumps on The Nature Conservancy fee-owned, conservation easements, offices, and lands where The Nature Conservancy has a management lease or agreement. Utility data was collected for all properties from 2021 billing records. CO2e was calculated based on the carbon emitting activity data (i.e., gallons of propane) times a carbon emission factor per activity and the global warming potential (S2 Table) [26]. Replacing the use of grid-sourced electricity and burning of fossil fuels with solar panels and heat pumps is presumed to reduce the CO2e emissions completely.

## Results

**Fig 1:**
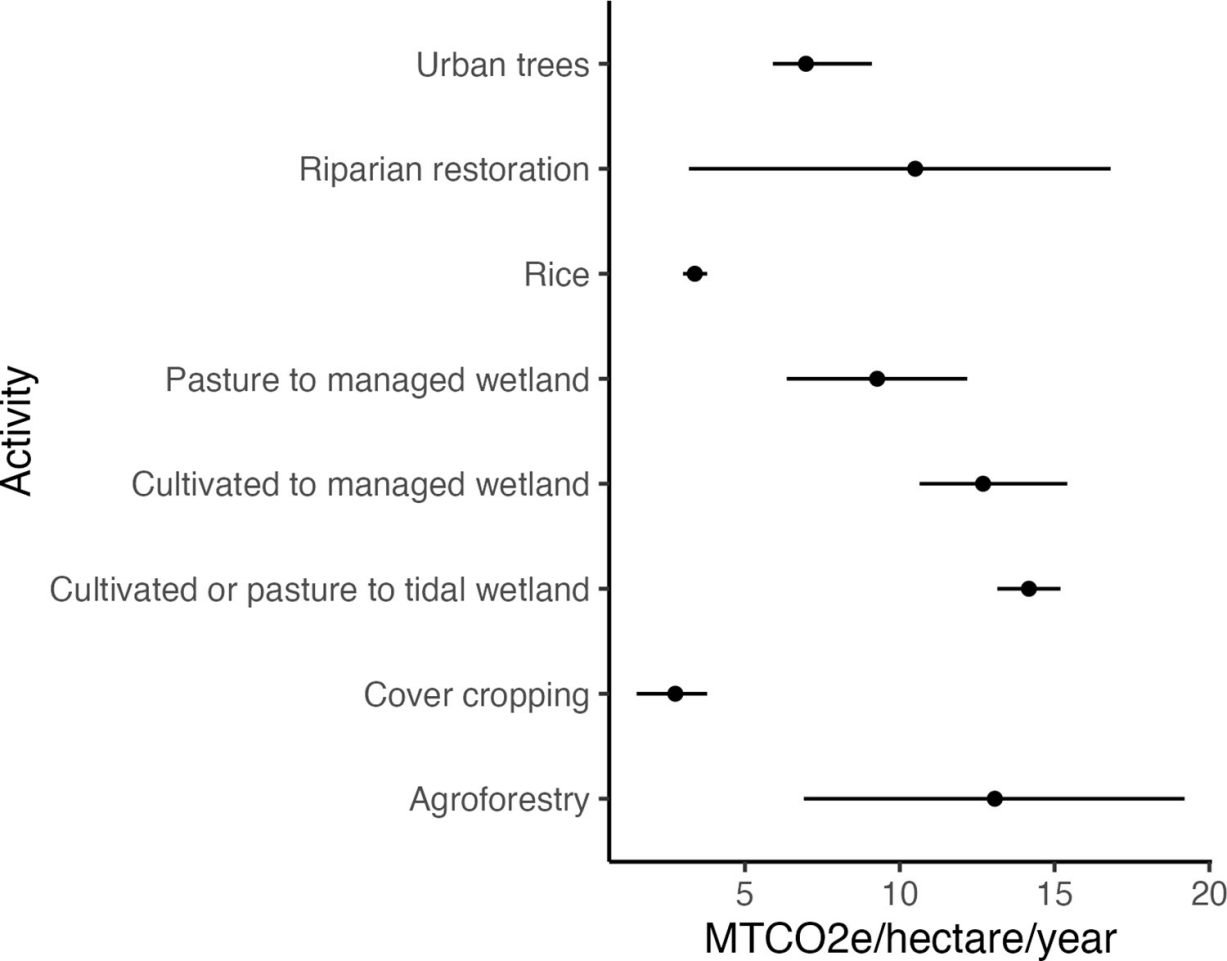
Sequestration rates for all NCS activities. Mean and ranges based on past studies (S1 Table).

**Fig 2:**
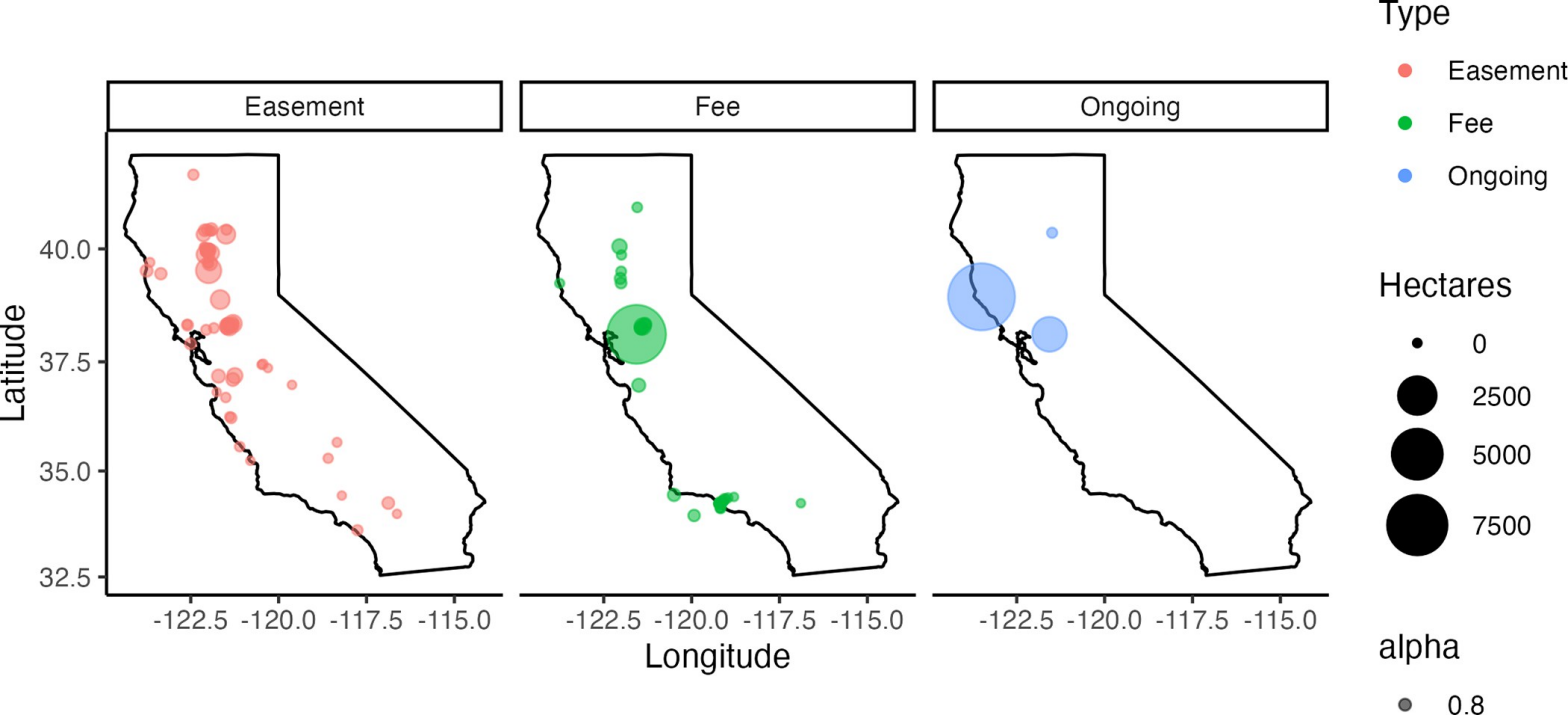
Potential NCS area for all activities combined on individual conservation easements and fee properties. Ongoing NCS activities are occurring at the Garcia River Forest (largest area), Childs Meadow, and Staten Island.

Based on the literature review, the NCS activity with the highest sequestration rate was *tidal wetland restoration*, with a mean value of 14.2 MTCO2e ha –1 year –1 (Fig 1, S4 Table). The activity with the lowest sequestration rate was *cover cropping* with a mean value of 2.75 MTCO2e ha –1 year –1. All NCS activities stored on average 9.5 MTCO2e ha –1 year –1. Potential NCS activities were identified across 37 fee properties covering 7,842 ha, or 11% of all fee-owned land (Fig 2). This total area is close to the goal of NCS activities being implemented on 10% of fee lands in California by 2030. NCS activities on conservations easements were identified across 65 properties covering a smaller total area, 3,149 ha. Clean energy transitions were identified for 14 properties (Table 3).

**Table 3:** Utility emission reduction potential. The greatest potential CO2e reduction from switching to clean energy is at the Staten Island property where water pumps are run to keep the island from going underwater due to a long history of land subsidence from farming the Delta.

TNC Property	Type	2021 Total CO2e
		(MTCO2e year^-1^)
Staten Island	Fee	437.1
Cosumnes River Preserve	Lease	18.12
Santa Cruz Island Preserve	Fee	17.53
Dangermond Preserve	Fee	14.78
Sacramento Office	Fee	7.24
Dye Creek Preserve	Management Agreement	5.96
Randall Tehachapi Preserve	Fee	5.25
Llano Seco Rancho	Easement	3.09
Sacramento River	Fee	1.80
Big Bear	Fee	1.70
Santa Clara River	Fee	1.00
Landreth Preserve	Fee	0.87
Spindrift Point	Fee	0.62
Sacramento River	Management Agreement	0.45

**Fig 3:**
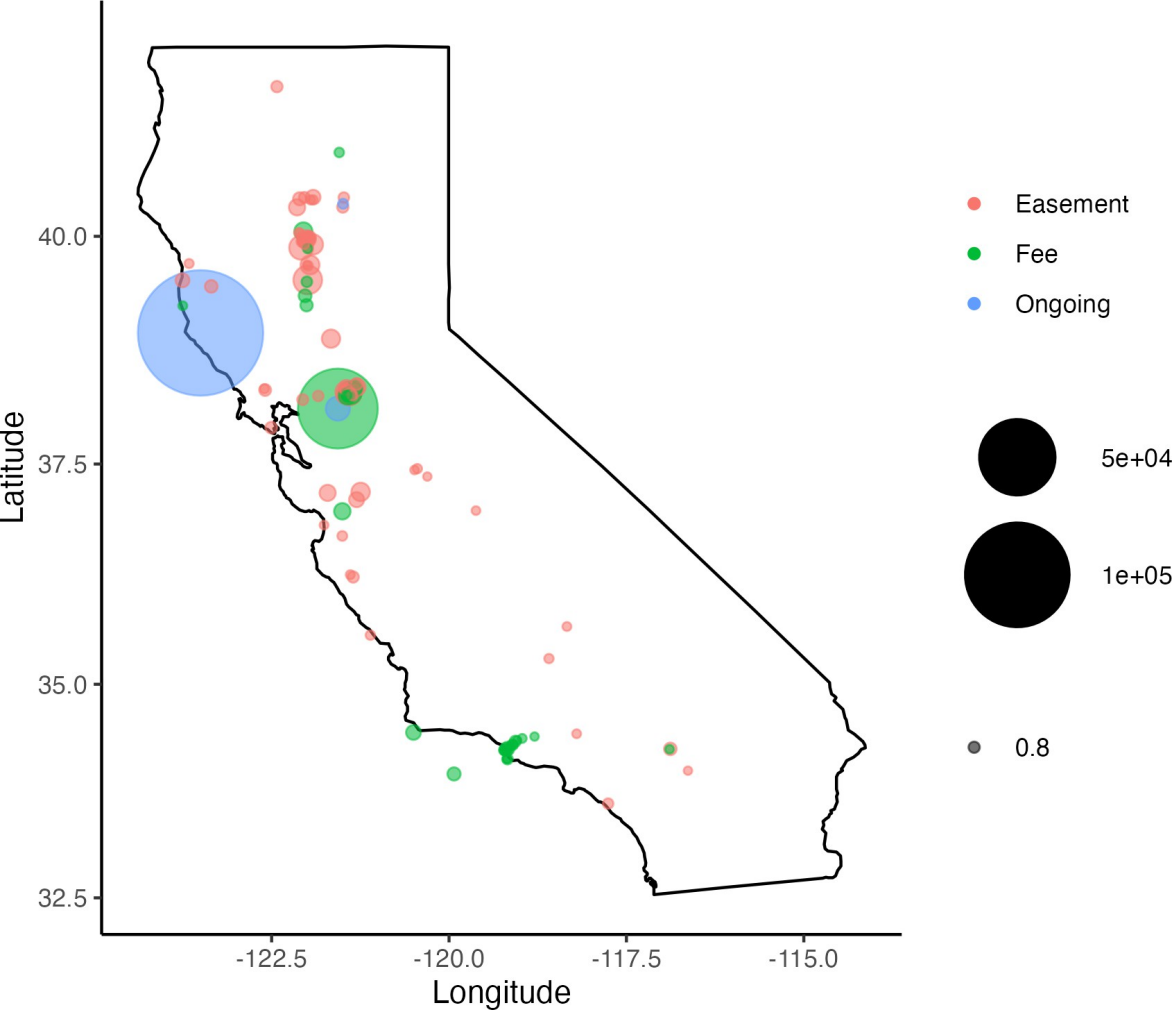
CO2e reduction potential of NCS activities and clean energy on The Nature Conservancy’s conservation easements, fee-owned properties, and ongoing projects in California. The largest blue circle highlights the CO2e reduction occurring at the Garcia River Forest property. Staten Island shows ongoing CO2e reduction from conversion of corn to rice and wetland restoration (blue) inside the potential CO2e reduction (green) if most of the property was cover cropped only.

The total climate mitigation potential of all the NCS activities and clean energy solutions was 63,663 MTCO2e year –1 on fee lands and 27,091 MTCO2e year –1 on conservation easements (Fig 3). Clean energy accounted for 516 MTCO2e year –1 across fee lands and other properties with 85% of the total reduction potential from water pumps at the Staten Island property. The CO2e reduction potential on fee properties is equivalent to 15,152 gasoline-powered cars driven for one year or 8,302 homes’ energy use for one year [27]. The annual reduction potential of all properties combined was less than the ongoing annual sequestration at the Garcia River Forest conservation easement from *changes to forest management* (147,749 MTCO2e year –1) alone. Implementing all potential NCS and clean energy solutions on fee properties would not reach the organization’s 2030 goal and represents 3% of the state’s CO2e reduction goal. Whereas reduction from the Garcia River Forest represents 7% of the state’s CO2e reduction goal.

**Fig 4:**
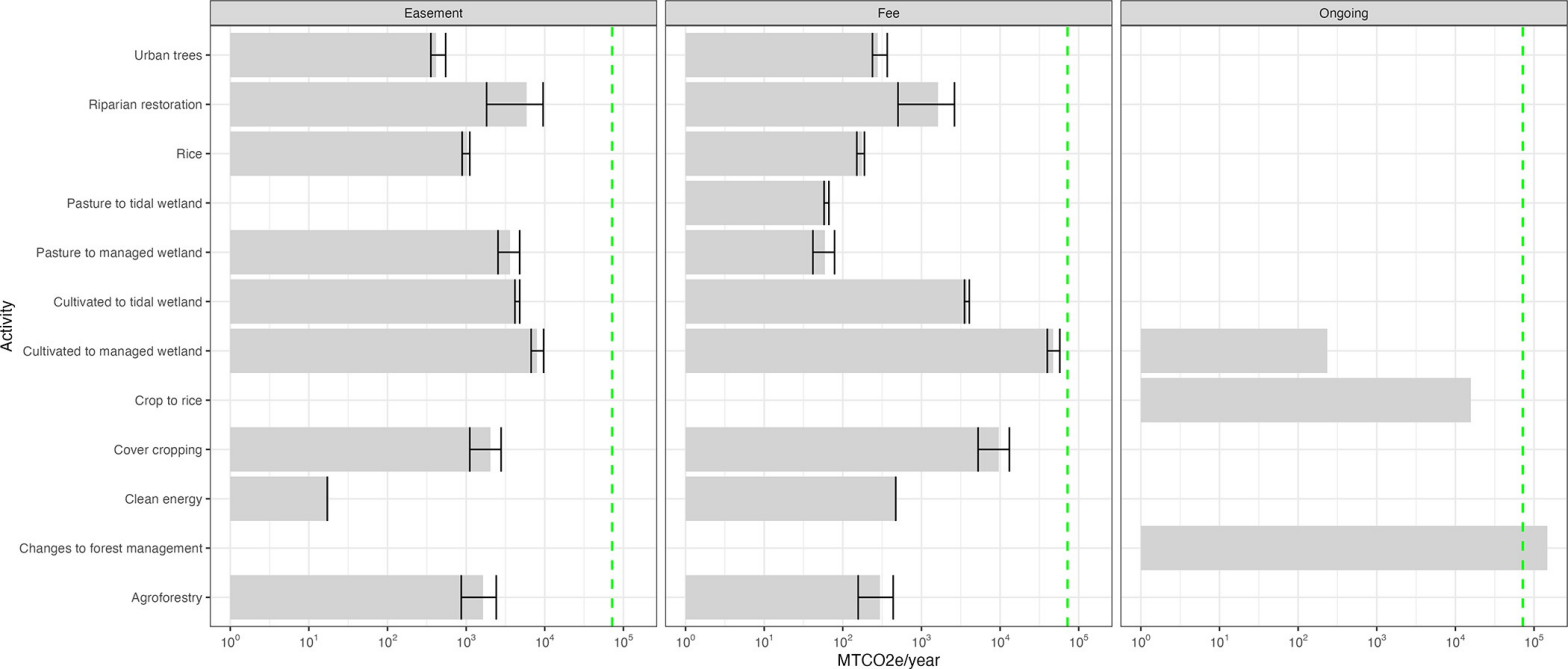
Mean and range of annual CO2e reduction from potential NCS activities and ongoing projects. The dashed green line shows The Nature Conservancy’s annual reduction goal of 72,000 MTCO2e per year by 2030. Note the log-scale on the x-axis.

The activity with the greatest climate mitigation potential was the same for fee-owned properties and conservation easements (Fig 4). On all fee properties together, *cultivated to managed wetland* had the greatest average reduction potential (47,277 MTCO2e year –1) followed by *cover cropping* (9,624 MTCO2e year –1). On all conservation easement properties together, *cultivated to managed wetland* (7,958 MTCO2e year –1) was the activity with the greatest reduction potential followed by *riparian restoration* (5,880 MTCO2e year –1). The area available for these three NCS activities was more than the other activities. *Cultivated to managed wetland restoration* and *cover cropping* covered 48% and 45% respectively of the total area identified for NCS on fee properties. On conservation easements the area identified for NCS was distributed more evenly across different activities: *cover cropping* (24%), *cultivated to managed wetland* (20%), *riparian restoration* (18%), *pasture to managed wetland* (13%), and the other NCS were less than 10% of the total area.

One fee owned property, Staten Island, had the largest climate mitigation potential for restoration, agricultural management, and clean energy transition (55,328 MTCO2e year –1), but the identified NCS would overlap in space. The reduction potential at the 3,723-ha property included four activities: *Cover cropping*, *cultivated to managed wetland restoration*, *rice best management practices*, and *clean energy* transition. *Wetland restoration* was identified for 3,596 ha, almost the entire property, which would leave less area for *cover cropping* and *rice best management practices*. The Nature Conservancy, California chapter decided to restore a small area of wetland (176 ha) and convert corn to rice (1,671 ha) at Staten Island. This blend of two NCS activities across a portion of the property will account for 11,180 MTCO2e year^-1^ once fully implemented, which represents 25% of the potential reduction from *cultivated to managed wetland* (45,315 MTCO2e year^-1^) only on the property. Across all properties, seven had overlapping wetland restoration and other NCS activities. If only wetland restoration or agricultural NCS activities were implemented, this non-overlapping total represented 96% of the total including overlapping activities, indicating the overestimate had a small impact on the overall CO2e reduction potential.

## Discussion

The CO2e reduction potential of restoration, agricultural management, and clean energy transitions on all fee-owned properties (63,663 MTCO2e year –1) would not reach the goal of 72,000 MTCO_2_e year –1 set for 10% of The Nature Conservancy’s fee-owned properties in California. One explanation is that the 2030 goal had a high sequestration rate (10 MTCO2e ha –1 year –1) compared to the mean values reported in the literature for cover cropping (1.50–3.78 MTCO2e ha –1 year –1) which represented almost half of the total area identified for NCS on fee properties. Another explanation is that new studies on sequestration rates were initiated in 2015 when the Cap-and-Trade market began funding NCS in California, and these studies had lower sequestration rates for some NCS activities. In this study, *tidal wetland restoration* sequestration was less than in an earlier study in California published before the Cap-and-Trade market started (mean 14 and 21 MTCO2e ha –1 year –1, respectively) [3].

Additionally, the main land cover type is annual grassland on fee properties where only one feasible NCS activity applied, *wetland restoration* of former peatlands. If *compost application* became feasible for The Nature Conservancy or there were sequestration rates available for *woodland restoration*, both of which would apply to annual grasslands, it is likely that total CO2e reduction potential would be greater and closer to the goal. The elimination of compost application because of potential biodiversity impacts limited the CO2e reduction potential. There are many calls to consider how feasibility will constrain the implementation of NCS [28–30], but few studies that directly address feasibility from the landowner or smallholder farmer’s perspective [31, 32] or expert perspectives at larger geographical scales [33–35]. The need for more information on sequestration rates for NCS activities and the long-term climate mitigation potential is also a high research priority [36].

To reach the 2030 goal, the chapter could implement NCS on conservation easements. This is challenging because of the need to have multiple landowners grant permission to implement NCS. *Wetland restoration* and *cover cropping* on agricultural lands were the most promising activities on easements, but some question whether NCS activities on agricultural lands will scale-up given it will require large numbers of individual owners to implement and maintain the activities over multiple decades [37]. Additionally, there are likely tradeoffs with agricultural production that could limit adoption of *wetland restoration* and other NCS. There is a need for more information on the feasibility of implementing NCS on easements, specifically the acceptability of different NCS and potential tradeoffs for landowners as these are likely be different than the acceptability and tradeoffs found for The Nature Conservancy.

This study focused on restoration and agricultural management NCS activities on current properties but did not account for the impact of land protection, which is likely The Nature Conservancy’s biggest contribution to climate mitigation [5, 6]. By preventing land conversion and implementing *changes to forest management* by default when setting easement terms for industrial timberland, The Nature Conservancy is protecting biodiversity and carbon stocks. The Conservation Fund is claiming the carbon credit for *changes to forest management* at the Garcia River Forest as the landowner, so The Nature Conservancy does not count this activity towards it CO2e reduction goal. However, future land deals where The Nature Conservancy holds the fee title and is the landowner could provide an opportunity for not only avoided conversion of carbon rich habitat but also other high impact NCS like *changes to forest management*.

*Changes to forest management* on the Garcia River Forest easement was the NCS and property with the highest mitigation impact, more than three times the potential of all other NCS activities combined and contributes substantially to the state’s reduction goal. This finding matches past studies that found changes to forest management had the largest mitigation potential in California [3] and Oregon [4]. This highlights the potential for the two largest private landowners in California, both industrial timber operators, to have a disproportionate impact on carbon mitigation if they implement or continue to implement *changes to forest management* that are additive. However, Marvin et al. [5] found that if you include avoided loss of soil carbon storage, then land conservation becomes the NCS with the largest mitigation potential in California. The biggest impact the organization can have will likely be from land protection first, changing forest or agricultural management second, and restoring ecosystems third [6].

Most of the reduction potential from restoration, agricultural management, and clean energy transition was concentrated in a single property. At Staten Island there was overlap in the area identified for potential wetland restoration and agricultural management, representing a tradeoff if only one activity was pursued at the expense of the other. By choosing to implement a mixture of some wetland restoration and some conversion of corn to rice, The Nature Conservancy did not maximize climate mitigation but balanced the need to support sandhill cranes that feed on the leftover corn post-harvest, maintain a working farm, and sequester carbon in the restored wetland and converted rice fields. Implementing wetland restoration only at Staten would provide four times greater climate mitigation than the current mix of activities, but wetland restoration would have eliminated the leftover corn supply for the cranes conflicting with the conservation objectives defined for the property.

Clean energy solutions on The Nature Conservancy’s fee-owned lands contributed modestly to CO2e reduction compared to NCS. While we accounted for the electricity and fossil fuel emissions from buildings and water pumps on the properties, this is not a complete accounting of the chapter’s carbon footprint. Most of the carbon emissions pre-Covid pandemic were due to air travel by staff and transportation to and from offices (unpublished data). Reducing these indirect emissions will be key to mitigating The Nature Conservancy’s carbon footprint. Adopting a portfolio of different NCS activities and clean energy solutions is likely they best past forward for The Nature Conservancy to mitigate climate change and follows the state’s carbon neutrality plan [10] and recommendations to focus on both NCS and clean energy to mitigate climate change [38].

The Nature Conservancy, California chapter is not the only organization setting ambitious CO2e reduction goals based on the implementation of NCS and clean energy. Other state and country chapters of The Nature Conservancy have set reduction goals that combined add up to 3 GTCO_2_e year –1 (3 billion metric tons) by 2030. Conservation International set a goal of avoiding 2 GTCO_2_e year –1 by conserving carbon rich ecosystems, specifically peat, mangroves, and old-growth forests. U.S. Agency for International Development’s (USAID) goal is 6 GTCO_2_e year –1 by conserving, restoring, and managing 100 M ha and implementing clean energy. The combined reduction goal of these three organizations is 11 GTCO_2_e year –1, almost half the estimated global potential of NCS [2].

While ambitious goals are needed to hold the increase in average temperature to 2° C over pre-industrial levels per the Paris Agreement, adoption and implementation may be slower than hoped for. This may be due to landowners’ acceptance of different NCS activities or needing to balance tradeoffs with other land uses such as biodiversity conservation and food production as discussed in this study. Adoption may also be slow because carbon market payments are too low, NCS do not qualify for payments, or there are questions about the long-term security of the funding [39]. There is also a time-lag to implement NCS due to planning and permitting requirements for restoration or delayed CO2e reduction impact post-restoration [6]. The Nature Conservancy and other large private landowners are in a unique position to implement a diverse NCS and clean energy portfolio, document the co-benefits and tradeoffs, and inspire more widespread adoption by other private landowners.

Limitations in this study include not accounting for the costs, incomplete accounting of indirect emissions, and not incorporating the risk of loss. Costs could be incorporated into a future study to guide The Nature Conservancy’s investments in NCS solutions that have the greatest return on investment. Future studies could also consider indirect emissions from cattle grazing, fertilizer application, fuel powered vehicles, and other activities by lessees or property owners on the properties. There is also the issue of permanence in climate mitigation from NCS that may be at risk from climate change and increases in wildfire and drought reversing the reduction. Incorporating risk of loss under climate change into the analysis of reduction could inform where to prioritize NCS activities with the greatest likelihood of success now and in the future under climate change.

## Conclusions

This study demonstrates how CO2e reduction from NCS and clean energy transitions can be assessed for an individual large landowner. We show how the potential CO2e reduction from future implementation of restoration, changes to agricultural management, and switching to clean energy on fee-owned properties in California will not reach the chapter’s 2030 CO2e goal.

The Nature Conservancy, California chapter would need to consider implementing NCS on properties with conservation easements or identifying future land protection deals with carbon rich ecosystems or high impact NCS to reach their goal. This finding is consistent with emerging science suggesting that land conservation and changes to forest management have a bigger climate mitigation impact than other NCS activities. The chapter is also balancing tradeoffs between maximizing climate mitigation and biodiversity conservation objectives, as illustrated at the Staten Island property. Our work provides a simple and generalizable approach to estimating CO2e reduction from feasible NCS and clean energy transitions that large landowner’s can use to understand the climate mitigation potential of their lands.

## Supporting information

S1 Table Stewardship and science team survey. The survey addressed whether it was feasible, and it made sense to implement an individual NCS activity on The Nature Conservancy, California fee or conservation easement properties. Scores were: (1) strongly disagree, (2) disagree, (3) neither agree nor disagree, (4) agree, and (5) strongly agree.
(DOCX)

S2 Table Emission factors used to calculate clean energy CO2e reduction potential. (DOCX)

S3 Table Search terms for the literature review. Science Direct was the second review and we did not include repeat articles from the Google Search.
(DOCX)

S4 Table Sequestration rates based on past studies. Studies that reported MgC per hectare were converted using the equation: MgC/ha x (44g/mole CO2)/(12 g/mole C) = MTCO2e/ha. We used low and high sequestration rates based on the low mean and high mean when multiple studies available for an activity. When only one study available the standard error or 95% confidence interval used to represent the low and high sequestration rate.
(DOCX)

S1 Fig Feasibility survey. (DOCX)
