# Supplementary material for: Climate mitigation potential of natural climate solutions and clean energy on The Nature Conservancy properties in California, USA
Source: PLoS One. 2024 Oct 21;19(10):e0311195. doi: 10.1371/journal.pone.0311195 (PMC11493287; doi:10.1371/journal.pone.0311195)
Supplement: S1 Table — The survey addressed whether it was feasible, and it made sense to implement an individual NCS activity on The Nature Conservancy, California fee or conservation easement properties. Scores were: (1) strongly disagree, (2) disagree, (3) neither agree nor disagree, (4) agree, and (5) strongly agree. (DOCX) [file pone.0311195.s001.docx]

**S1 Table. Stewardship and science team survey**. The survey addressed whether it was feasible and it made sense to implement an individual NCS activity on The Nature Conservancy, California fee or conservation easement properties. Scores were: (1) strongly disagree, (2) disagree, (3) neither agree nor disagree, (4) agree, and (5) strongly agree.

| Activity | Mean score (1-5) | Min | Max | Responses (n) | Activity conducted on TNC property? |
| --- | --- | --- | --- | --- | --- |
| Wetland restoration | 4.8 | 4 | 5 | 12 | Yes |
| Riparian restoration | 4.7 | 4 | 5 | 14 | Yes |
| Cover cropping | 4.5 | 4 | 5 | 11 | Yes |
| Woodland restoration | 4.2 | 3 | 5 | 15 | Yes |
| Agroforestry | 4.0 | 4 | 4 | 15 | No |
| Urban tree reforestation | 3.8 | 3 | 5 | 13 | Yes |
| Rice BMPs | 3.7 | 1 | 5 | 9 | Yes |
| Compost application | 2.8 | 2 | 4 | 13 | No |
