# Supplementary material for: Climate mitigation potential of natural climate solutions and clean energy on The Nature Conservancy properties in California, USA
Source: PLoS One. 2024 Oct 21;19(10):e0311195. doi: 10.1371/journal.pone.0311195 (PMC11493287; doi:10.1371/journal.pone.0311195)
Supplement: S2 Table — (DOCX) [file pone.0311195.s002.docx]

**S2 Table. Emission factors used to calculate clean energy CO2e reduction potential**.

| Utility Type | Category | GHG | Emission Factor | Description | Source |
| --- | --- | --- | --- | --- | --- |
| Electricity | Power generation | CO_2_e | 515.483 lb/MWh | CO2 equivalent total output emission rate | EPA 2022^c^ |
|  | T&D^a^ | CO_2_e | 0.1123 kg/kWh | Adjustment for Transmission and Distribution Losses Induced Emissions | IEA 2019^d^ |
|  | WTT^b^ generation | CO_2_e | 0.1066 kg/kWh | DEFRA 2021 – WTT – UK & Overseas Electricity | GOV.UK 2021^e^ |
|  | WTT T&D | CO_2_e | .0058 kg/kWh |  |  |
| Propane | Direct Emissions | CO_2_ | 5.72 kg/gallon | Emission Factors for Greenhouse Gas Inventories - Table 1 Stationary Combustion | EPA 2020^f^ |
|  |  | CH_4_ | 0.27 g/gallon |  |  |
|  |  | N_2_O | 0.05 g/gallon |  |  |
|  | WTT | CO_2_e | 0.2532 kg/liter | DEFRA 2021 – WTT Fuels | GOV.UK 2021 |

^a^ T&D = Transmission and Distribution Losses

^b^ WTT = Well-to-Tank

^c^ United States Environmental Protection Agency (EPA). 2022. *Emissions & Generation Resource Integrated Database (eGRID)* Washington DC: Office of Atmospheric Protection, Clean Air Markets Division.

^d^ International Energy Agency (IEA). 2019. Emissions Factors database. https://www.iea.org/data-and-statistics/data-product/emissions-factors-2022#documentation

^e^ United Kingdom. 2021. Government conversion factors for company reporting of greenhouse gas emissions.

^f^ United States Environmental Protection Agency (EPA). 2020. Emission Factors for Greenhouse Gas Inventories. https://www.epa.gov/sites/default/files/2021-04/documents/emission-factors_mar2020.pdf
