# Supplementary material for: Climate mitigation potential of natural climate solutions and clean energy on The Nature Conservancy properties in California, USA
Source: PLoS One. 2024 Oct 21;19(10):e0311195. doi: 10.1371/journal.pone.0311195 (PMC11493287; doi:10.1371/journal.pone.0311195)
Supplement: S3 Table — Science Direct was the second review and we did not include repeat articles from the Google Search. (DOCX) [file pone.0311195.s003.docx]

**S3 Table. Search terms for the literature review.** Science Direct was the second review and we did not include repeat articles from the Google Search.

| Database | Main search string | Second part of search string | # articles reviewed | # of articles included | Date accessed |
| --- | --- | --- | --- | --- | --- |
| Google Scholar | "sequestration” AND "California" | AND "agroforestry" | 50 | 1 | November 14, 2023 |
|  |  | AND "cover crop" | 50 | 2 |  |
|  |  | AND "rice" | 50 | 1 |  |
|  |  | AND "riparian" | 50 | 2 |  |
|  |  | AND "urban tree" | 50 | 1 |  |
|  |  | AND "wetland" | 50 | 4 |  |
|  |  | AND “tidal wetland” | 50 | 1 |  |
| Google Scholar | “carbon” AND “California” | AND "agroforestry" | 50 | 0 |  |
|  |  | AND "cover crop" | 50 | 1 |  |
|  |  | AND "rice" | 50 | 0 |  |
|  |  | AND "riparian" | 50 | 2 |  |
|  |  | AND "urban tree" | 50 | 0 |  |
|  |  | AND "wetland" | 50 | 2 |  |
|  |  | AND “tidal wetland” | 50 | 0 |  |
| Science Direct | “carbon” AND “California” | AND "agroforestry" | 11 | 0 | November 15, 2023 |
|  |  | AND "cover crop" | 50 | 0 |  |
|  |  | AND “rice” | 50 | 0 |  |
|  |  | AND "riparian" | 26 | 0 |  |
|  |  | AND "urban tree" | 14 | 0 |  |
|  |  | AND "wetland" | 51 | 2 |  |
|  |  | AND “tidal wetland” | 18 | 1 |  |
| Science Direct | "sequestration” AND "California" | AND "agroforestry" | 50 | 0 |  |
|  |  | AND "cover crop" | 50 | 0 |  |
|  |  | AND "rice" | 50 | 0 |  |
|  |  | AND "riparian" | 50 | 0 |  |
|  |  | AND "urban tree" | 50 | 0 |  |
|  |  | AND "wetland" | 50 | 0 |  |
|  |  | AND “tidal wetland” | 50 | 0 |  |
