# Supplementary material for: Climate mitigation potential of natural climate solutions and clean energy on The Nature Conservancy properties in California, USA
Source: PLoS One. 2024 Oct 21;19(10):e0311195. doi: 10.1371/journal.pone.0311195 (PMC11493287; doi:10.1371/journal.pone.0311195)
Supplement: S4 Table — Studies that reported MgC per hectare were converted using the equation: MgC/ha x (44g/mole CO2)/(12 g/mole C) = MTCO2e/ha. We used low and high sequestration rates based on the low mean and high mean when multiple studies available for an activity. When only one study available the standard error or 95% confidence interval used to represent the low and high sequestration rate. (DOCX) [file pone.0311195.s004.docx]

**S4 Table. Sequestration rates based on past studies.** Studies that reported MgC per hectare were converted using the equation: MgC/ha x (44g/mole CO2)/(12 g/mole C) = MTCO2e/ha. We used low and high sequestration rates based on the low mean and high mean when multiple studies available for an activity. When only one study available the standard error or 95% confidence interval used to represent the low and high sequestration rate.

| # | **NCS** | **Source** | **MgC/ha/yr** | **MTCO2e/ha/year** | | | | | | **Notes** |
| --- | --- | --- | --- | --- | --- | --- | --- | --- | --- | --- |
|  |  |  | Mean | Mean | | Range of means | SE | 95% CI | |  |
| 1 | agroforestry | Fargione et al. 2018 | 3.56 | 13.07 | **13.07** |  |  | **6.9** | **19.2** | Outside CA |
| 2 | cover cropping | [White et al. 2020](https://journals.plos.org/plosone/article?id=10.1371/journal.pone.0228677) | 1.03 | 3.78 | **2.75** | **1.50- 3.78** |  |  |  | System 1, no compost, cover crops only (mean 8.3 MgC/ha)/8 years |
| 3 |  | [Mitchell et al. 2015](https://acsess.onlinelibrary.wiley.com/doi/full/10.2134/agronj14.0415) | 0.81 | 2.96 |  |  |  |  |  | Conventional tillage cover crop treatment, soil carbon only (26.36-19.91 MgC/ha)/8 year |
| 4 |  | [Wolff et al. 2018](https://www.sciencedirect.com/science/article/pii/S0167198717301228) | 0.41 | 1.50 |  |  |  |  |  | No tillage cover crop |
| 5 | rice | Fargione et al. 2018 | 0.92 | 3.38 |  |  |  | **3.00** | **3.78** | Outside CA |
| 6 | riparian restoration | [Matzek et al. 2020](https://link.springer.com/article/10.1186/s13021-020-00150-7) | 0.87 | 3.19 | **10.50** | **3.19- 16.81** |  |  |  | Floodplain soil carbon |
| 7 |  | [Matzek et al. 2020](https://link.springer.com/article/10.1186/s13021-020-00150-7) | 1.12 | 4.11 |  |  |  |  |  | Upper bank soil carbon |
| 8 |  | [Dybala et al. 2018](https://besjournals.onlinelibrary.wiley.com/doi/pdfdirect/10.1111/1365-2664.13272) | 2.94 | 10.79 |  |  |  |  |  | Biomass and soil carbon planted (2.29+0.65 MgC/ha/year) |
| 9 |  | [Matzek et al. 2015](https://onlinelibrary.wiley.com/doi/full/10.1111/avsc.12400) | 3.67 | 13.47 |  |  |  |  |  |  |
| 10 |  | [Dybala et al. 2018](https://besjournals.onlinelibrary.wiley.com/doi/pdfdirect/10.1111/1365-2664.13272) | 3.98 | 14.61 |  |  |  |  |  | Biomass and soil carbon naturally regenerating(3.59+0.39 MgC/ha/year) |
| 11 |  | [Matzek et al. 2018](https://onlinelibrary.wiley.com/doi/full/10.1111/avsc.12400) | 4.58 | 16.81 |  |  |  |  |  | High end of carbon sequestration used to represent riparian woodland values similar to the NCS activity in this study. The rate is not linear |
| 13 | cultivated to managed wetland | [Arias-Ortiz et al. 2021](https://agupubs.onlinelibrary.wiley.com/doi/pdf/10.1029/2021JG006573) | 2.9 | 10.64 | **12.69** | **10.64- 15.41** |  |  |  | Young wetland |
| 14 |  | [Arias-Ortiz et al. 2021](https://agupubs.onlinelibrary.wiley.com/doi/pdf/10.1029/2021JG006573) | 3.2 | 11.74 |  |  |  |  |  | Old wetland |
| 15 |  | [Hemes et al. 2019](https://www.sciencedirect.com/science/article/pii/S0168192319300176?via%3Dihub) | 2.90 | 10.64 |  |  |  |  |  | East End |
| 16 |  | Maziarz et al. 2019 | 4.20 | 15.41 |  |  |  |  |  | Not clear if cultivated |
| 17 |  | [Hemes et al. 2019](https://www.sciencedirect.com/science/article/pii/S0168192319300176?via%3Dihub) | 4.09 | 15.01 |  |  |  |  |  | West Pond |
| 18 | pasture to managed wetland | [Hemes et al. 2019](https://www.sciencedirect.com/science/article/pii/S0168192319300176?via%3Dihub) | 1.73 | 6.35 | **9.27** | **6.35- 12.18** |  |  |  | Mayberry, Sherman Island, Table 1 |
| 19 |  | [Reed et al. 2022](https://esajournals.onlinelibrary.wiley.com/doi/pdfdirect/10.1002/eap.2677?casa_token=hRglE5KYVUwAAAAA:GDOMCufJgtLNN2rj8tM34DWSpmv7B1u-odRzc39b8nNzaZ_N-2wczlYYyv64BwYkyLioDgEG1CrjD5XExg) | 3.32 | 12.18 |  |  |  |  |  | Root carbon plus soil carbon |
| 20 | tidal wetland restoration | [Arias-Ortiz et al. 2021](https://agupubs.onlinelibrary.wiley.com/doi/pdf/10.1029/2021JG006573) | 3.86 | 14.17 | **14.17** |  | **1.02** |  |  | Pasture to young wetland |
| 21 | urban tree | Fargione et al. 2018 | 1.9 | 6.97 | **6.97** |  |  | **5.9** | **9.1** |  |

References

Arias‐Ortiz, A., Oikawa, P.Y., Carlin, J., Masqué, P., Shahan, J., Kanneg, S., Paytan, A. and Baldocchi, D.D., 2021. Tidal and nontidal marsh restoration: a trade‐off between carbon sequestration, methane emissions, and soil accretion. *Journal of Geophysical Research: Biogeosciences*, *126*(12), p.e2021JG006573.

Dybala, K.E., Steger, K., Walsh, R.G., Smart, D.R., Gardali, T. and Seavy, N.E., 2019. Optimizing carbon storage and biodiversity co‐benefits in reforested riparian zones. *Journal of Applied Ecology*, *56*(2), pp.343-353.

Hemes, K.S., Chamberlain, S.D., Eichelmann, E., Knox, S.H. and Baldocchi, D.D., 2018. A biogeochemical compromise: The high methane cost of sequestering carbon in restored wetlands. *Geophysical Research Letters*, *45*(12), pp.6081-6091.

Hemes KS, Chamberlain SD, Eichelmann E, Anthony T, Valach AC, Kasak K, et al. 2019. Assessing the carbon and climate benefit of restoring degraded agricultural peat soils to managed wetlands. Agric For Meteorol, 268(1), 202–214.

Matzek, V., Stella, J. and Ropion, P., 2018. Development of a carbon calculator tool for riparian forest restoration. *Applied vegetation science*, *21*(4), pp.584-594.

Matzek, V., Lewis, D., O’Geen, A., Lennox, M., Hogan, S.D., Feirer, S.T., Eviner, V. and Tate, K.W., 2020. Increases in soil and woody biomass carbon stocks as a result of rangeland riparian restoration. *Carbon balance and management*, *15*, pp.1-15.

Maziarz, J., Vourlitis, G.L. and Kristan, W., 2019. Carbon and nitrogen storage of constructed and natural freshwater wetlands in southern California. *Ecological Engineering*, *142*, p.100008.

Mitchell, J.P., Shrestha, A., Horwath, W.R., Southard, R.J., Madden, N., Veenstra, J. and Munk, D.S., 2015. Tillage and cover cropping affect crop yields and soil carbon in the San Joaquin Valley, California. Agronomy Journal, 107(2), pp.588-596.

Silver, W.L., Ryals, R. and Eviner, V., 2010. Soil carbon pools in California’s annual grassland ecosystems. *Rangeland Ecology & Management*, *63*(1), pp.128-136.

White, K.E., Brennan, E.B., Cavigelli, M.A. and Smith, R.F., 2020. Winter cover crops increase readily decomposable soil carbon, but compost drives total soil carbon during eight years of intensive, organic vegetable production in California. *PLoS One*, *15*(2), p.e0228677.

Wolff, M.W., Alsina, M.M., Stockert, C.M., Khalsa, S.D.S. and Smart, D.R., 2018. Minimum tillage of a cover crop lowers net GWP and sequesters soil carbon in a California vineyard. *Soil and Tillage Research*, *175*, pp.244-254.
