# Supplementary material for: Climate mitigation potential of natural climate solutions and clean energy on The Nature Conservancy properties in California, USA
Source: PLoS One. 2024 Oct 21;19(10):e0311195. doi: 10.1371/journal.pone.0311195 (PMC11493287; doi:10.1371/journal.pone.0311195)
Supplement: S1 Fig — (DOCX) [file pone.0311195.s005.docx]

FEASIBILITY ASSESSMENT AND PAST IMPLEMENTATION

We want to understand what activities you think are feasible to implement on The Nature Conservancy fee and easement lands in California. We will describe 10 activities and then ask you if you think it makes sense for TNC to implement that activity (Question #1). This is hypothetical and does not imply that TNC will implement an activity.

We also want to understand if an activity was already implemented on our fee lands (Question #2).

We thank you for you participation in advance.

***All your answers will be treated confidentially, and the results will not contain any information that can be used to identify you.

# Agricultural activities (5 total)

- **Agroforestry** – plant trees along field boundaries (Central Valley only)
- **Compost application** – Add organic and mineral soil amendments to grasslands (Chaparral and Oak Woodland Ecoregions)
- **Cover cropping** – plow under cover crop prior to cash crop planting
- **Fertilizer reduction** – Nitrogen application, type
- **Rice cultivation BMP** – farming practices

### **Agroforestry** – plant trees along field boundaries (Central Valley only)

## I think agroforestry could make sense to implement on some TNC fee lands.

ENTER YOUR ANSWER IN THE CHAT.

1. Strongly disagree
2. Disagree
3. Neither agree nor disagree
4. Agree
5. Strongly agree

Was agroforestry already implemented on TNC fee land?

Please type in chat the property name and we will follow up.

**Compost application** – Add organic and mineral soil amendments to grasslands

### (Chaparral and Oak Woodland Ecoregions)

I think compost application could make sense to implement on some TNC fee lands.

1. Strongly disagree
2. Disagree
3. Neither agree nor disagree
4. Agree
5. Strongly agree

Was compost application already implemented on TNC fee land?

Please type in chat the property name and we will follow up.

**Cover cropping** – plow under cover crop prior to cash crop planting

## I think cover cropping could make sense to implement on some TNC fee lands.

1. Strongly disagree
2. Disagree
3. Neither agree nor disagree
4. Agree
5. Strongly agree

Was cover cropping already implemented on TNC fee land?

Please type in chat the property name and we will follow up.

**Fertilizer reduction –** Nitrogen application, type

I think fertilizer reduction could make sense to implement on some TNC fee lands.

1. Strongly disagree
2. Disagree
3. Neither agree nor disagree
4. Agree
5. Strongly agree

Was fertilizer reduction already implemented on TNC fee land?

Please type in chat the property name and we will follow up.

**Rice cultivation BMPs** – farming practices

I think rice cultivation BMPs could make sense to implement on some TNC lands.

1. Strongly disagree
2. Disagree
3. Neither agree nor disagree
4. Agree
5. Strongly agree

Was rice cultivation

# BMPs already implemented on TNC fee land?

Please type in chat the property name and we will follow up.

# Tree focused activities (4 total)

- - **Riparian restoration** – establishing forest cover along streams (ag and grassland regions)
  - **Urban tree reforestation** – planting trees
  - **Woodland restoration** – planting native hardwoods in areas where removed or lost (Oak Woodland and Chaparral Ecoregion only)
  - **Reforestation post-wildfire** –

replanting trees in areas burned at high severity last 2010-2020

**Riparian restoration** – establishing forest cover along streams (ag and grassland regions)

I think riparian restoration could make sense to implement on some TNC fee lands.

1. Strongly disagree
2. Disagree
3. Neither agree nor disagree
4. Agree
5. Strongly agree

Was riparian restoration already implemented on TNC fee land?

Please type in chat the property name and we will follow up.

**Urban tree reforestation** – planting trees

I think urban tree reforestation could make sense to implement on some TNC fee lands.

1. Strongly disagree
2. Disagree
3. Neither agree nor disagree
4. Agree
5. Strongly agree

Was urban tree

reforestation already implemented on TNC fee land?

Please type in chat the property name and we will follow up.

### **Woodland restoration** – planting native hardwoods in areas where removed or lost (Oak Woodland and Chaparral Ecoregion only)

I think woodland restoration could make sense to implement on some TNC fee lands.

1. Strongly disagree
2. Disagree
3. Neither agree nor disagree
4. Agree
5. Strongly agree

Was woodland

restoration already implemented on TNC fee land?

Please type in chat the property name and we will follow up.

**Reforestation post-wildfire** – replanting trees in areas burned at high severity last 2010-2020

I think reforestation post-wildfire could make sense to implement on some TNC lands.

1. Strongly disagree
2. Disagree
3. Neither agree nor disagree
4. Agree
5. Strongly agree

Was reforestation post-

## wildfire already implemented on TNC fee land?

Please type in chat the property name and we will follow up.

# Wetland Restoration (1 Activity)

- - **Cultivated to tidal wetland**
  - **Cultivated to wetland**
  - **Pasture to wetland**
  - **Pasture to tidal wetland**
- From peatland sites previously converted
- Irrigated pasture only

**Wetland restoration** – cultivated peatland

I think wetland restoration could make sense to implement on some TNC lands.

1. Strongly disagree
2. Disagree
3. Neither agree nor disagree
4. Agree
5. Strongly agree

Was wetland restoration already implemented on TNC fee land?

Please type in chat the property name and we will follow up.
